# Pandemics and Human Capital: Panel Data Evidence From 122 Countries

**DOI:** 10.3389/fpubh.2022.885957

**Published:** 2022-04-25

**Authors:** Jianmin Sun, Kaihao Cai, Hongzhou Shen

**Affiliations:** ^1^School of Management, Nanjing University of Posts and Telecommunications, Nanjing, China; ^2^International Monetary Fund, Washington, DC, United States

**Keywords:** COVID-19 pandemic, pandemics uncertainty, human capital stocks, World Pandemics Uncertainty index, panel data studies

## Abstract

This study investigates the determinants of human capital stocks, measured by the Penn World Table data, in the panel dataset of 122 countries from 1996 to 2019. The special role is given to the World Pandemics Uncertainty index to measure pandemics uncertainty across countries. The paper finds that per capita gross domestic product and population increase human capital stocks. The decline in fertility rates leads to a higher level of human capital. The interesting evidence is that pandemics' uncertainty decreases human capital. These findings are valid when we focus on both the high-income and the middle/low-income economies. These results are against the Becker-Lewis theory's validity since sources of uncertainty are negatively related to human capital.

## Introduction

How does pandemic related uncertainty affect human capital? There are different answers to this question. Firstly, one should expect a negative relationship. Human capital investments are the lifetime spending for gaining knowledge and skills to improve children's potential and increase economic performance in developing and developed economies. The progress in human capital has significantly declined due to the COVID-19 pandemic (1). Specifically, the COVID-19 pandemic has negatively affected developing economies' education and health outcomes [see, e.g., (2)]. However, according to the World Bank (1), more than one billion children have been negatively affected by school closures, and this issue has distorted human capital earnings and learning. The study also shows a significant decline in health services for children, especially in developing countries. In an earlier study, Percoco (3) shows that the Spanish flu between 2018 and 2020 caused a decline in the schooling rates in the Italian regions, where they were negatively affected by the pandemic. Dash et al. (4) and Deng et al. (5) also observe the negative effects of the COVID-19 pandemic on human capital stocks in different countries.

Secondly, according to the Becker-Lewis theory, living in more uncertain economic conditions (e.g., pandemics periods) can increase investments in human capital as uncertainty leads to a decline in fertility (6). Thus, there will be an increase in human capital since parents will focus on fewer children in their lifetime. For instance, Galor (7, 8), Galor and Weil (9), Kalemli-Ozcan (10), Kimura and Yasui (11), and Lagerlöf (12) also empirically show that uncertainty increases the average returns of human capital investments. Therefore, the Becker-Lewis theory suggests a positive association between uncertainty and human capital investments (13).

However, several papers show no significant relationship between uncertainty and human capital investments, especially in developing economies due to the role of the informal economy [see, e.g., (14, 15)]. These studies also show that uncertainty leads to a lower fertility rate. Still, the effect of uncertainty on human capital is negligible or ambiguous.

Overall it is unclear how can pandemic related uncertainty can affect human capital in both developed and developing countries in the long run. At this juncture, this paper investigates the determinants of human capital stocks in the panel dataset across 122 countries from 1996 to 2019. Our human capital measure comes from the human capital index, defined in the Penn World Table data of Feenstra et al. (16). It is constructed by Barro and Lee (17) average years of schooling data. At this stage, the special role is given to the World Pandemics Uncertainty index to measure pandemics uncertainty across countries.

Our paper shows that per capita gross domestic product and population increase human capital stocks. However, the decline in fertility rates leads to a higher human capital level. Our novel finding is that pandemics related uncertainty decreases the human capital. These findings remain valid when we focus on both the high-income and the middle/low-income economies. Therefore, our results are against the Becker-Lewis theory's validity since sources of uncertainty are negatively related to human capital stocks.

The rest of the paper is organized as follows. Section Data and Empirical Model explains the data and the empirical model. Section Empirical Results discusses the empirical results and Section Conclusion concludes.

## Data and Empirical Model

This paper uses the panel dataset of 122 countries from 1996 to 2019.^1^ The data frequency is annual, and the beginning date of 1996 is related to the availability of the panel data sample. In addition, following the spirit in Chen et al. (18), we also divide the sample into the high-income and the middle-income/low-income economies. The country classification is based on the income calculations in the World Bank (19). Then, this paper estimates the following equation:


(1) $$\begin{array}{r} HCI_{i,t}=\gamma_{0}+\gamma_{1}\;WPI_{i,t}+{\gamma_{2}\;X}_{i,t}+\;\varphi_{i,t}\;{+\varepsilon }_{i,t} \end{array}$$

In Equation (1), *HCI*_*i, t*_, is the human capital index, *WPI*_*i, t*_ is the World Pandemics Uncertainty index. *X*_*i, t*_ denotes the controls in the estimations. Countries are represented by *i*, and periods are tagged by *t*. In addition, φ_*i, t*_ is the fixed-effects for countries and periods. Note that ε_*i, t*_is error terms. The traditional method, i.e., fixed-effects, is used to estimate this model.

The dependent variable is the human capital index, which captures knowledge and skills across countries. The index is defined in the Penn World Table (PWT) (version 10) by Feenstra et al. (16), and it is constructed by Barro and Lee's (17) average years of schooling data. The income level is measured by the log per capita gross domestic product (constant USD prices). The log total population captures country size. These data are downloaded from Feenstra et al. (16). Total fertility rate (births per woman) is also added to the models, and the data are obtained from the World Bank (20). The fertility rate addresses the Becker-Lewis theory, which shows the trade-off between children's quality and quantity. We expect the positive effects of income and the country's size on human capital stocks. Besides, the fertility rate should be negatively related to the human capital index.

On the other hand, the main variable of interest is the World Pandemic Uncertainty index. The related data are introduced by Ahir et al. (21). This index uses texting mining techniques using the country reports of the Economist Intelligence Unit dataset (22). The text searches are based on findings of words, such as “pandemics” and “uncertainty”, in the country reports of the Economist Intelligence Unit. According to Ahir et al. (21), the World Pandemic Uncertainty index significantly varies across the countries and provides uncertainty shocks related to pandemics. At this stage, Table 1 reports descriptive statistics of the variables in the estimations.

**Table 1:** Descriptive statistics.

**Variable**	**HCI**	**LnGDPC**	**POP**	**FERT**	**WPI**
Mean	2.448	11.66	2.649	3.105	2.978
Maximum	4.351	16.85	7.268	7.716	438.9
Minimum	1.053	6.266	−0.648	0.977	0.000
Standard deviation	0.716	1.840	1.373	1.656	17.50
Observation	2,928	3,216	3,216	3,174	3,312

***Source:*** *The authors' estimations*.

The pairwise correlations are also reported in Table 2.

**Table 2:** Pairwise correlations.

**Variable**	**HCI**	**LnGDPC**	**POP**	**FERT**	**WPI**
HCI	1.000				
LnGDPC	0.523	1.000			
POP	0.048	0.728	1.000		
FERT	−0.805	−0.568	−0.051	1.000	
WPI	−0.097	−0.069	0.009	0.061	1.000

***Source:*** *The authors' estimations*.

Table 2 shows that human capital is positively related to per capita gross domestic product and total population. However, the correlation of the World Pandemics Uncertainty index and the fertility rate with human capital is negative. Still, the correlation between the per capita income and the World Pandemics Uncertainty index is negative. The correlations between the total population and fertility with the human capital are negative. The pairwise correlations are in line with the theoretical expectations.

## Empirical Results

Table 3 reports the findings of the panel unit root test of Pesaran (23) for each variable in the estimation.

**Table 3:** Panel unit root test results.

**Concept**	**Levels**		**First Differences**		**Decision**
**Variable**	**Intercept**	**Intercept and trend**	**Intercept**	**Intercept and trend**	
HCI	−8.347 [0.000]	−4.024 [0.000]	−21.57 [0.000]	−13.38 [0.000]	I(0)
LnGDPC	−5.553 [0.000]	−3.001 [0.001]	−13.23 [0.000]	−9.524 [0.000]	I(0)
POP	−21.27 [0.000]	−20.63 [0.000]	−19.32 [0.000]	−14.34 [0.000]	I(0)
FERT	−17.10 [0.000]	−18.67 [0.000]	−14.62 [0.000]	−9.801 [0.000]	I(0)
WPI	−7.393 [0.000]	−4.506 [0.000]	−26.26 [0.000]	−20.73 [0.000]	I(0)

*Probability values are in the brackets*.

***Source:*** *The authors' estimations*.

The null hypothesis of this unit root test is that the series follows the unit root process. We provide the results for the constant and the trend terms. All results show that the null hypothesis of the unit root has been rejected, and therefore, the series is stationary. Next, we move on to the fixed-effects estimations.

Table 4 reports the findings of the fixed-effects estimations to show the effects of pandemic related uncertainty on human capital investments in the panel dataset of 122 countries from 1996 to 2019.

**Table 4:** Panel data fixed-effects estimation results.

**Variables**	**All countries**	**All countries**	**High-income**	**High-income**	**Middle and low income**	**Middle and low income**
LnGDPC_t_	0.286*** (0.005)	0.264*** (0.006)	0.462*** (0.010)	0.459*** (0.011)	0.266*** (0.006)	0.228*** (0.007)
POP_t_	0.035** (0.016)	0.033** (0.016)	0.013** (0.006)	0.013** (0.006)	0.043** (0.018)	0.037** (0.017)
FERT_t_	–	−0.025*** (0.007)	–	−0.023*** (0.008)	–	−0.052*** (0.008)
WPI_t_	−0.024** (0.012)	−0.025** (0.011)	−0.073*** (0.028)	−0.074*** (0.028)	−0.030*** (0.010)	−0.031*** (0.010)
Constant term	−0.940*** (0.059)	−0.609*** (0.089)	−2.975*** (0.134)	−2.892*** (0.138)	−0.855*** (0.064)	−0.238** (0.105)
Observations	2,928	2,806	1,016	982	1,912	1,824
Number of countries	122	122	42	42	80	80
Adjusted R-squared	0.553	0.562	0.748	0.757	0.538	0.557

The dependent variable is the index for human capital investments (HCI). The standard errors are in ( ). * and ** represent the 1 and 5% significance levels, respectively.

***Source:*** The authors' estimations. ***p < 0.01, **p < 0.05, *p < 0.10.

Columns 1 and 2 provide the findings for 122 countries from 1996 to 2019. Besides, the results for 42 high-income economies are reported in Columns 3 and 4. Furthermore, the findings for middle-income and low-income economies are provided in Columns 5 and 6.

In terms of results for all countries, the World Pandemics Uncertainty (WPI) index coefficients are around −0.024, and they are significant at the 5% level. Similarly, the World Pandemics Uncertainty index coefficients are around −0.074 for the high-income economies. They are significant at the 1% level. In addition, the coefficients of the World Pandemics Uncertainty index are around −0.031 for the middle-income and the low-income economies, and they are significant at the 1% level. These findings align with the previous findings of Dash et al. (4) and Deng et al. (5), Percoco (3) and the World Bank (1). However, these results are against the Becker-Lewis theory's validity since sources of uncertainty are negatively related to human capital. These findings show that the pandemic-related uncertainty adversely affects human capital in developing and developed countries.

In terms of the control variables, there are significant effects of the controls on the human capital investments (*HCI*). It is observed that the per capita gross domestic product (l*nGDPC*) increases the human capital stocks. The related coefficients are statistically significant at the 1% level. The population (*POP*) is positively related to human capital investments. The related coefficients are also statistically significant at the 5% level. However, the fertility rates (*FERT*) decrease the human capital stocks as expected. The related coefficients are also statistically significant at the 1% level. The effects of control variables on the human capital are in line with the theoretical expectations of Becker-Lewis theory and the growth dynamics of Galor (8).

## Conclusion

This paper examined the determinants of human capital stocks, measured by the Penn World Table data, in the panel dataset of 122 countries from 1996 to 2019. A special role is given to the World Pandemics Uncertainty index, provided by Ahir et al. (21), to measure pandemics uncertainty across countries. We found that per capita gross domestic product and population increase human capital stocks. However, the decline in fertility rates leads to a higher human capital level. Our novel evidence is that pandemics' uncertainty decreases human capital. These findings are valid when we focused on both the high-income and the middle/low-income economies. Our results are against the Becker-Lewis theory's validity since sources of uncertainty are negatively related to human capital.

Finally, it is essential to note that our panel data samples are limited to evaluate cross-country variations. Therefore, future papers should focus on the country sample using the micro-level data. The data can also be updated until 2021 to understand the dynamics of the COVID-19 pandemic and forecast the effects of post-pandemic uncertainty shocks on different measures of human capital stocks.

## Data Availability Statement

The original contributions presented in the study are included in the article/supplementary material, further inquiries can be directed to the corresponding author/s.

## Author Contributions

JS writing the manuscript and data collection. KC writing the manuscript and estimations. HS writing and reviewing the manuscript. All authors contributed to the article and approved the submitted version.

## Funding

The authors acknowledge the financial support from the Natural Science Foundation of China (Grant no: 71974102) and the Philosophy and Social Science Fund of Tianjin City, China (Grant no: TJYJ20-012).

## Conflict of Interest

The authors declare that the research was conducted in the absence of any commercial or financial relationships that could be construed as a potential conflict of interest.

## Publisher's Note

All claims expressed in this article are solely those of the authors and do not necessarily represent those of their affiliated organizations, or those of the publisher, the editors and the reviewers. Any product that may be evaluated in this article, or claim that may be made by its manufacturer, is not guaranteed or endorsed by the publisher.

## Footnotes

^1^Albania, Algeria, Angola, Argentina, Armenia, Australia, Austria, Bangladesh, Belgium, Benin, Bolivia, Botswana, Brazil, Bulgaria, Burkina Faso, Burundi, Cambodia, Cameroon, Canada, Central African Republic, Chile, China, Colombia, Congo DR, Congo Republic, Costa Rica, Côte d'Ivoire, Croatia, Czech Republic, Denmark, Dominican Republic, Ecuador, Egypt, El Salvador, Ethiopia, Finland, France, Gabon, the Gambia, Germany, Ghana, Greece, Guatemala, Honduras, Hungary, India, Indonesia, Iran, Iraq, Ireland, Israel, Italy, Jamaica, Japan, Jordan, Kazakhstan, Kenya, Korea Republic, Kuwait, Kyrgyz Republic, Laos, Latvia, Lesotho, Liberia, Lithuania, Madagascar, Malawi, Malaysia, Mali, Mauritania, Mexico, Moldova, Mongolia, Morocco, Mozambique, Myanmar, Namibia, Nepal, the Netherlands, New Zealand, Nicaragua, Niger, Nigeria, Norway, Pakistan, Panama, Paraguay, Peru, the Philippines, Poland, Portugal, Qatar, Romania, Russia, Rwanda, Saudi Arabia, Senegal, Sierra Leone, Singapore, Slovak Republic, Slovenia, South Africa, Spain, Sri Lanka, Sudan, Sweden, Switzerland, Tajikistan, Tanzania, Thailand, Togo, Tunisia, Turkey, Uganda, Ukraine, the United Arab Emirates, the United Kingdom, the United States, Uruguay, Vietnam, Zambia, and Zimbabwe.
